# Metabolic Syndrome and Male Infertility: The Role of Obesity and Chronic Inflammation

**DOI:** 10.3390/biomedicines14081739

**Published:** 2026-08-01

**Authors:** Shreeram Behera, Koushik Bhattacharya, Soumya Jal, Gopal Krishna Purohit, Ashok Kumar Sah, Shagun Agarwal, Rasha Babiker, Ashwani Bhardwaj, Ayman Husein Mohamed Alfeel

**Affiliations:** 1 School of Allied and Healthcare Sciences, Centurion University of Technology and Management, Khurda Road, Jatni 752050, Odisha, Indiasoumya.4787@gmail.com (S.J.); 2 Herrick Healthcare, Bhubaneswar 751013, Odisha, India; gopalpurohit@hereditybio.in; 3 Department of Medical Laboratory Sciences, College of Applied and Health Sciences, A’ Sharqiyah University, Ibra 415, Oman; 4 School of Allied Health Sciences, Galgotias University, Greater Noida 203201, Uttar Pradesh, India; shagunmpt@gmail.com; 5 Department of Physiology, RAK College of Medical Sciences, Ras Al Khaimah Medical and Health Sciences University, Ras Al Khaimah 11172, United Arab Emirates; 6 Department of Physiology, Faculty of Medicine and Surgery, National University—Sudan (NUSU), Khartoum 11115, Sudan; 7 Department of Medical Laboratory Technology, Kalpana Chawala Govt. Polytechnic for Women, Ambala City 134003, Haryana, India; bhardwaj.ashwani58@gmail.com; 8 Department Medical Laboratory Sciences, College of Health and Applied Sciences, Gulf Medical University, Ajman 4184, United Arab Emirates; dr.aymanhussein@gmc.ac.ae

**Keywords:** metabolic syndrome, male infertility, obesity, chronic inflammation, oxidative stress, insulin resistance, spermatogenesis, adipokines

## Abstract

Metabolic syndrome (MetS) is a complex metabolic disorder that includes central fat accumulation and insulin resistance, abnormal lipid levels, high blood pressure, and ongoing mild inflammation as its main symptoms. The global rise in metabolic syndrome parallels the increasing prevalence of male infertility, suggesting a potential association between metabolic health and reproductive function. Male fertility suffers from obesity, which represents a major element of metabolic syndrome because it disrupts hormonal balance, raises scrotal temperatures, and causes oxidative damage and HPG axis malfunction. Excess body fat functions as an endocrine system that induces the body to produce pro-inflammatory cytokines, which include tumor necrosis factor-α and interleukin-6, and various other inflammatory substances that result in ongoing body-wide inflammation. The state of inflammation leads to damaged spermatogenesis, which results in lower testosterone production and impaired semen quality that includes changes in sperm concentration, motility, morphology, and DNA integrity. The combination of insulin resistance and metabolic disturbances that occur in MetS leads to oxidative stress and mitochondrial dysfunction, which harms male reproductive abilities in testicular tissue. Recent research shows that adipokines and endocrine disruptors, together with epigenetic modifications, function as mediators that establish the connection between metabolic syndrome and male infertility. The development of targeted therapeutic strategies and lifestyle interventions requires researchers to study how obesity and chronic inflammation interact with each other to create reproductive dysfunction. This review defines the pathophysiological mechanisms that connect metabolic syndrome to male infertility by focusing on how obesity-related inflammation affects male reproductive health.

## 1. Introduction

The increasing prevalence of male infertility has become a worldwide medical issue that contributes to the total reproductive health problems faced by all nations. However, male-related factors cause approximately 40 to 50 percent of all infertility cases [1]. Male reproductive health has experienced a substantial decline during the last 50 years, which has resulted in decreased sperm concentration, disrupted sperm motility, abnormal sperm shape, and heightened sperm DNA damage. Multiple environmental, genetic, lifestyle, and metabolic elements have been identified as causes of this decline, with metabolic disorders receiving more attention than other factors. The increasing cases of metabolic syndrome (MetS) have been demonstrated to directly affect the decline of male reproductive abilities [2,3]. The disorder known as metabolic syndrome involves multiple metabolic pathways that lead to central obesity, insulin resistance, dyslipidemia, hypertension, and disturbed glucose metabolism.

The global prevalence of MetS has reached new heights because people now lead inactive lives and consume unhealthy food, and cities have expanded [4]. The metabolic disturbances that occur in this condition create health risks, which include cardiovascular diseases and type 2 diabetes, while they also produce major hormonal changes that affect both endocrine and reproductive systems. Research shows that men with metabolic syndrome develop hormonal imbalances that result in testicular dysfunction and decreased semen production, thus demonstrating how metabolic health affects their ability to conceive [5,6].

Obesity and metabolic syndrome are not only associated with impaired reproductive physiology but also substantially increase the clinical risk of male infertility. Epidemiological studies have consistently demonstrated that overweight and obese men exhibit a higher prevalence of infertility, hypogonadism, and abnormal semen parameters than men with normal body weight. Large population-based investigations indicate that obesity is associated with approximately a 20–30% higher risk of infertility, whereas severe obesity (BMI ≥ 35 kg/m^2^) may increase this risk by up to 40–80%, depending on the study population and infertility definition. Similarly, metabolic syndrome has been linked to significantly reduced sperm concentration, progressive motility, normal morphology, and increased sperm DNA fragmentation. A recent meta-analysis including more than 13,000 participants reported that men with metabolic syndrome exhibited significantly lower sperm count, sperm concentration, total and progressive motility, and normal morphology, together with increased DNA fragmentation compared with metabolically healthy controls. Clinical cohort studies have further demonstrated that men with metabolic syndrome have a markedly greater prevalence of testosterone deficiency, reduced sex hormone-binding globulin concentrations, and obesity-associated hypogonadism, all of which contribute to impaired fertility. Importantly, the risk increases progressively with the number of metabolic syndrome components present, suggesting a cumulative detrimental effect of central obesity, insulin resistance, dyslipidemia, hypertension, and chronic inflammation on male reproductive function. These epidemiological findings complement the mechanistic evidence discussed throughout this review and emphasize that metabolic syndrome represents a clinically important and potentially modifiable risk factor for male infertility.

The metabolic syndrome includes several components, but obesity stands out as the main element that reduces male reproductive ability. Body fat functions as a distinct endocrine system that produces various bioactive substances such as adipokines, cytokines, and inflammatory mediators [7]. Obesity causes the hypothalamic–pituitary–gonadal HPG axis to malfunction, which results in lower testosterone levels and higher conversion rates of androgens to estrogens and disrupted spermatogenesis. The accumulation of fat in the scrotal area leads to higher testicular temperatures, which consequently hampers the processes of sperm production and development [8]. Chronic low-grade inflammation serves as an essential link that connects metabolic syndrome to male infertility. Obese people experience weight gain because their body stores fat in larger adipose cells, while their immune system sends white blood cells to their body, resulting in their body producing pro-inflammatory cytokines [9]. The body uses these inflammatory substances to generate oxidative stress, which creates a harmful environment for testicular cells while it destroys sperm through lipid peroxidation and DNA fragmentation [10]. Oxidative stress disrupts spermatozoa’s mitochondrial functions, which leads to lower sperm movement ability and decreases their capacity to fertilize eggs. The metabolic syndrome-associated insulin resistance creates two problems because it disrupts hormonal balance and decreases reproductive performance [11].

The review aims to summarize the current scientific evidence regarding how metabolic abnormalities influence hormonal regulation, spermatogenesis, and semen quality. The study intends to show the pathophysiological pathways through which obesity-related inflammatory responses, oxidative stress, and endocrine disruptions lead to male reproductive problems. The review summarizes new scientific studies that link oxidative stress, mitochondrial dysfunction, and epigenetic changes to reproductive disorders resulting from metabolic disorders [12].

## 2. Pathophysiological Mechanisms of Metabolic Syndrome

The pathophysiology of MetS involves a complex network of metabolic, hormonal, and inflammatory processes that interact to produce a cluster of metabolic abnormalities. People with metabolic syndrome experience three core symptoms, which include hyperglycemia, dyslipidemia, and hypertension, because their bodies develop persistent insulin resistance with compensatory hyperinsulinemia [13]. Oxidative stress functions as a key mechanism that drives both metabolic syndrome development and its associated health complications. The condition emerges when reactive oxygen species (ROS) production exceeds the capacity of the body’s antioxidant defense systems. The increased levels of ROS lead to cellular damage, which affects lipids, proteins, and DNA [14]. Oxidative stress in metabolic syndrome leads to three destructive pathways that cause inflammation, disrupt insulin signaling, and trigger mitochondrial impairment. The combined effects of these elements create worsened metabolic disturbances, which result in damage to bodily tissues. Endothelial dysfunction serves as a vital component that disrupts the normal operation of blood vessels within metabolic syndrome pathophysiology [15]. Endothelial cells function as crucial regulators that control vascular tone and blood flow and the body’s response to inflammation.

## 3. Obesity and Its Association with Metabolic Syndrome

Obesity serves as a major risk factor that directly leads to the development of MetS because it establishes the main characteristics of the disease. The condition occurs when people gain too much body fat, which results in metabolic problems and raises their risk for multiple chronic illnesses such as cardiovascular disease, type 2 diabetes, and reproductive system disorders [16]. The development of metabolic syndrome connects with obesity because it creates permanent low-level inflammation. The adipose tissue contains enlarged adipocytes that release pro-inflammatory cytokines that immune cells produce, including tumor necrosis factor-α (TNF-α), interleukin-6 (IL-6), and C-reactive protein (CRP). The body uses these inflammatory substances to disrupt normal metabolic processes, while their presence makes insulin resistance worse [17]. Obesity brings about medical issues that affect metabolic processes and endanger the normal functioning of endocrine and reproductive systems. Excess body fat creates a hormonal imbalance because it produces more estrogens through the conversion of androgens [18]. The hormonal imbalance that this condition causes in men leads to disruptions in the hypothalamic–pituitary–gonadal (HPG) axis together with decreased testosterone production and worsened spermatogenesis. The mechanisms that research studies use to examine the link between obesity and metabolic syndrome establish an essential connection to male infertility problems [19].

### 3.1. Types and Distribution of Adipose Tissue

Adipose tissue can be broadly classified into white adipose tissue (WAT), brown adipose tissue (BAT), and beige adipose tissue, each with distinct structural and functional characteristics. White adipose tissue, which is present in adults, constitutes the main type of adipose tissue because it stores excess energy as triglycerides. The endocrine organ functions through the secretion of adipokines, which include leptin, adiponectin, and resistin, and inflammatory cytokines [20]. The abdominal region shows excessive white adipose tissue accumulation, which doctors identify as a major risk factor for metabolic disorders that include obesity, insulin resistance, and metabolic syndrome [21]. Brown adipose tissue functions as a specialized tissue system that produces heat and consumes energy. The tissue achieves its heat production through its extensive mitochondrial network, which contains uncoupling protein-1 (UCP1). Infants possess greater quantities of brown adipose tissue than adults, who maintain small amounts in areas like their necks and supraclavicular regions and spinal areas [22].

Visceral adipose tissue exists as body fat that surrounds abdominal organs, including the liver, pancreas, and intestines. This fat type exists as a highly active metabolic substance that contributes to the development of metabolic syndrome. Visceral adipose tissue releases higher amounts of free fatty acids together with inflammatory cytokines and pro-inflammatory adipokines, which enter the bloodstream [23]. These substances cause insulin resistance, systemic inflammation, and dyslipidemia, increasing the risk of cardiovascular disease. The accumulation of visceral fat works as a primary indicator for metabolic syndrome because it closely connects with metabolic disruptions [24].

### 3.2. Hormonal Dysregulation in Obesity

The excessive fat buildup in obese individuals causes their bodies to lose control over essential signaling molecules, which generates hormonal imbalances that lead to metabolic syndrome and its related health problems. One of the most significant hormonal disturbances observed in obesity is insulin resistance. The condition causes body tissues, such as skeletal muscle, liver, and adipose tissue, to exhibit a decreased insulin response, leading to impaired glucose metabolism and uptake [25]. Obese people experience increased estrogen production because their body fat activates aromatase enzymes, which convert androgens into estrogens. The process results in higher estrogen production and lower testosterone levels, which causes hormonal imbalances that disrupt normal male reproductive functions [26].

### 3.3. Insulin Resistance and Metabolic Dysfunction

Insulin resistance constitutes the fundamental element of metabolic syndrome, which causes metabolic disorders and their related health problems to develop. Insulin enables cells to take in glucose, while it helps glycogen production and controls both fat and protein body processes [27]. Insulin resistance causes metabolic processes to malfunction, which results in body systems losing their ability to control both glucose and fat levels in the bloodstream. The body responds to insulin resistance by increasing insulin production from its pancreatic beta cells, which leads to hyperinsulinemia.

Excess adipose tissue serves as the primary factor that causes insulin resistance, especially when people gain excess visceral fat. The body stores visceral fat, which releases substantial quantities of free fatty acids that disrupt insulin signaling in both the liver and skeletal muscle. Elevated free fatty acids in the bloodstream increase hepatic glucose production, decrease glucose uptake by peripheral tissues, and promote liver triglyceride synthesis [28]. The metabolic disturbances create abnormal lipid profiles, which show increased triglyceride levels and decreased high-density lipoprotein (HDL) cholesterol levels that define metabolic syndrome [29].

The link between insulin resistance and abnormal lipid metabolism increases the likelihood of developing atherosclerosis and cardiovascular disease. Men who experience insulin resistance develop hormonal imbalances that disrupt their reproductive system functions, according to research about reproductive health. Insulin resistance increases oxidative stress in the testis, which results in damage to sperm cells that decreases their ability to move and stay alive [30].

### 3.4. Obesity-Induced Endocrine Alterations

**Figure 1:**
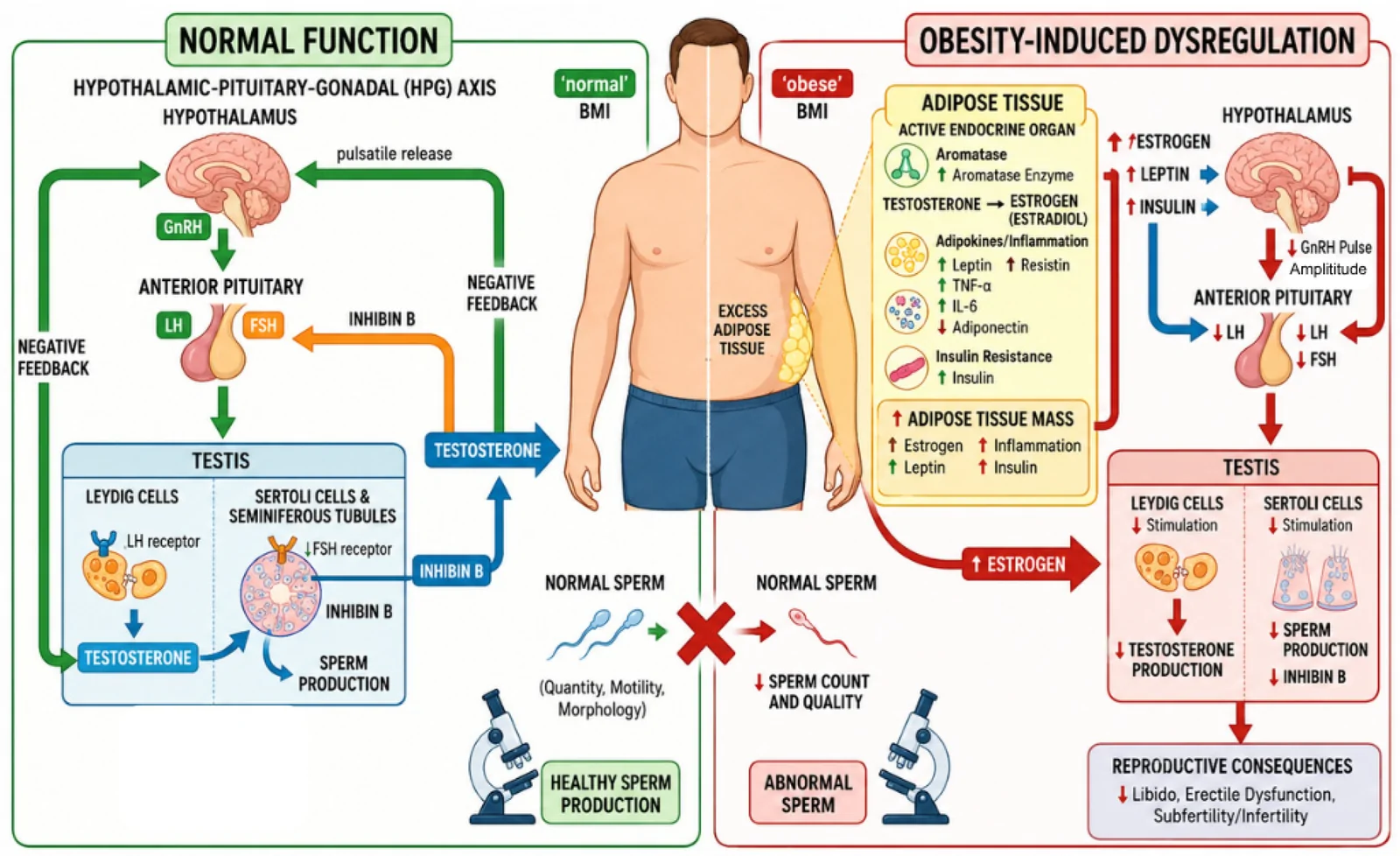
Schematic illustration of the molecular and endocrine mechanisms linking obesity and metabolic syndrome (MetS) with male infertility. The figure highlights the effects of insulin resistance, chronic inflammation, oxidative stress, hormonal dysregulation, and impaired Sertoli and Leydig cell function on spermatogenesis and semen quality.

Obesity leads to major changes in endocrine function that disrupt multiple hormonal pathways that control metabolism, energy balance, and reproductive processes. Adipose tissue functions as an active endocrine organ that releases a variety of hormones, cytokines, and adipokines when present in excessive quantities. The bioactive substances of this system control multiple body functions, which include appetite regulation, insulin sensitivity, inflammation, and hormone balance for reproduction. The growth and malfunction of adipose tissue in obese people cause their bodies to disrupt hormone production and control, which results in extensive endocrine system changes [31]. Obesity causes the body to release more pro-inflammatory cytokines, which include tumor necrosis factor-α and interleukin-6. The inflammatory mediators cause both chronic low-grade inflammation and disruption of endocrine signaling pathways (Figure 1). The body system faces disruptions because chronic inflammation prevents normal hormone production and signaling, which results in worsened metabolic and reproductive dysfunction [32]. Obesity affects the production and function of insulin, cortisol, and growth hormone, which control metabolic processes in the body. Insulin resistance leads to hyperinsulinemia, which results in changes to lipid metabolism and hormonal imbalance. Obese individuals experience decreased growth hormone secretion, which leads to weight gain through two processes: first, impaired lipid metabolism and second, increased fat storage [33].

This figure illustrates the impact of obesity on the hypothalamic–pituitary–gonadal (HPG) axis and male reproductive function. Under normal conditions, pulsatile GnRH secretion from the hypothalamus stimulates LH and FSH release from the anterior pituitary, which in turn regulate Leydig and Sertoli cell function to maintain testosterone production, inhibin B secretion, and normal spermatogenesis. In obesity, excess adipose tissue acts as an endocrine organ, increasing aromatase activity, estrogen production, leptin levels, insulin resistance, and inflammatory cytokines. These metabolic disturbances disrupt HPG-axis signaling by reducing GnRH pulsatility and altering gonadotropin secretion, leading to impaired Leydig and Sertoli cell activity. Consequently, testosterone production, inhibin B levels, and sperm production decline, resulting in abnormal semen parameters, sexual dysfunction, and reduced fertility potential.

## 4. Chronic Inflammation in Metabolic Disorders

Chronic inflammation exists as a fundamental characteristic that affects multiple metabolic diseases, including obesity and metabolic syndrome, type 2 diabetes mellitus, and cardiovascular diseases. The body uses acute inflammation as a short-term defense mechanism against both infections and injuries, while chronic inflammation establishes a continuous state of low-level inflammation that endures for multiple months or years [34]. The extended period of persistent inflammation disrupts normal metabolic balance, which results in metabolic disorders developing and worsening throughout time. The body experiences chronic inflammation because of its excess fat storage, particularly in visceral adipose tissue. The adipocytes in obese people experience cell growth, which leads to metabolic activation that drives them to produce different inflammatory substances and adipokines [35]. Chronic inflammation leads to the development of insulin resistance, which serves as the primary characteristic of metabolic syndrome. Pro-inflammatory cytokines cause disruptions to insulin receptor signaling pathways, which function in essential metabolic tissues including skeletal muscle, liver, and adipose tissue [36]. Prolonged exposure to ongoing inflammation together with insulin resistance leads to hyperglycemia, which progresses into type 2 diabetes.

### 4.1. Mechanisms of Low-Grade Chronic Inflammation

Chronic low-grade inflammation represents a continuous state of body-wide inflammation, which serves as a key factor that leads to obesity and metabolic syndrome, insulin resistance, and type 2 diabetes mellitus. Obesity-related adipose tissue expansion serves as the main factor that drives persistent low-grade chronic inflammation in humans [37]. Immune cells, including macrophages and T lymphocytes, enter adipose tissue to produce inflammatory cytokines, which include tumor necrosis factor-α (TNF-α), interleukin-6 (IL-6), and interleukin-1β (IL-1β) [38]. The body spreads these inflammatory substances through its bloodstream, which establishes a continuous state of inflammation that affects all body parts [39]. The second major mechanism describes how immune cells become activated, and macrophages undergo their polarization process. Macrophages in healthy adipose tissue maintain their anti-inflammatory M2 type phenotype, which enables them to restore damaged tissue and maintain proper metabolic functions. Obese people exhibit a transformation of their macrophages from their normal state to an active state, which produces inflammatory substances [40]. The M1-type macrophages produce excessive amounts of cytokines and chemokines, which strengthen inflammatory signaling pathways that result in widespread body inflammation. The shift in immune cell composition establishes a key mechanism that sustains chronic inflammatory conditions that develop from metabolic diseases. Chronic inflammation develops through the activation of intracellular pathways that signal inflammation. The body activates major molecular pathways, which include the nuclear factor kappa B (NF-κB) pathway and c-Jun N-terminal kinase (JNK) signaling pathway during times of metabolic stress and when they face inflammatory challenges. The signaling pathways enable cells to control the production of several inflammatory genes while they generate cytokines and chemokines, which maintain ongoing inflammatory processes [41].

### 4.2. Role of Pro-Inflammatory Cytokines and Adipokines

Pro-inflammatory cytokines and adipokines serve as essential factors that drive the onset and development of metabolic disorders, which specifically impact individuals with obesity and metabolic syndrome. The process of inflammation develops through the action of pro-inflammatory cytokines, which adipocytes and immune cells from the blood circulation produce inside adipose tissue [42]. The key cytokines that drive metabolic inflammation include TNF-α, interleukin-6 (IL-6), and interleukin-1β (IL-1β). These cytokines function as essential components that drive inflammatory signaling pathways while simultaneously disrupting standard metabolic control. The activity of TNF-α causes insulin resistance through its effects on insulin receptor function and subsequent signaling pathways that follow [43]. The production of C-reactive protein (CRP) by the liver is activated through IL-6, which leads to inflammation and worsens metabolic health problems [44]. The release of these cytokines from enlarged adipocytes and activated macrophages in obese individuals establishes an ongoing inflammatory state, which impacts multiple metabolic tissues. Elevated levels of pro-inflammatory cytokines cause three different disturbances, which include glucose metabolism impairment, lipid abnormalities, and endothelial dysfunction. These inflammatory mechanisms contribute to cardiovascular disease risk and exacerbate metabolic diseases characteristic of metabolic syndrome [45]. In addition, cytokines and adipokines secreted by adipose tissue represent a crucial factor in the regulation of metabolic activities and inflammatory response. The body uses different adipokines, which include resistin, visfatin, and chemerin, to mediate both inflammatory responses and metabolic changes. These molecules may promote inflammation, alter glucose metabolism, and contribute to the development of insulin resistance. The pro-inflammatory and anti-inflammatory adipokine imbalance between these two types of adipokines establishes the chronic inflammatory condition that exists in people with obesity and metabolic syndrome [46].

### 4.3. Oxidative Stress and Inflammatory Signaling Pathways

**Figure 2:**
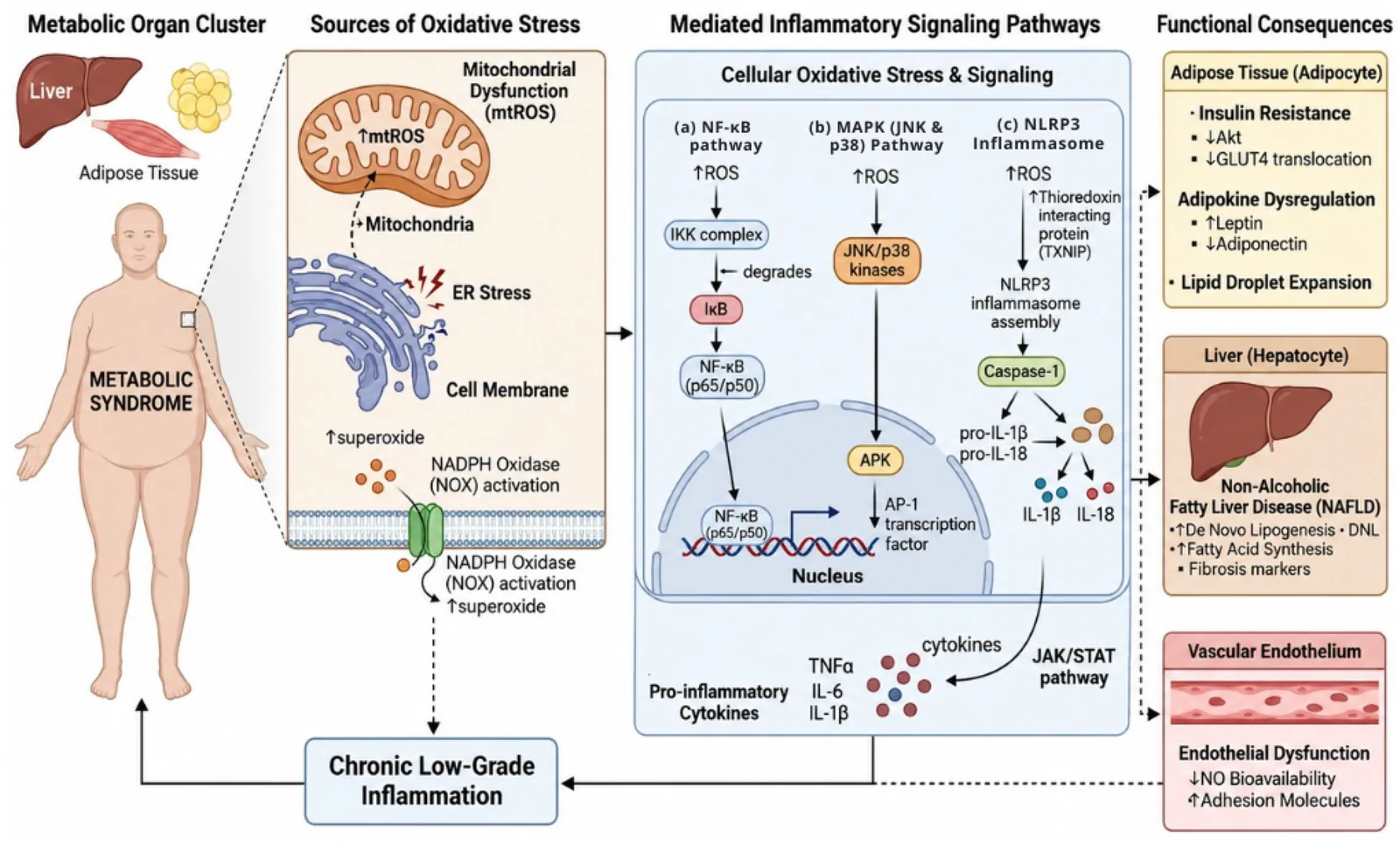
Oxidative stress-mediated inflammatory signaling pathways in metabolic syndrome.

The process of oxidative stress triggers metabolic diseases, which include obesity and metabolic syndrome, insulin resistance, and type 2 diabetes mellitus. Oxidative stress activates multiple pathways, but the nuclear factor kappa B (NF-κB) signaling pathway stands as the most essential pathway. The transcription factor NF-κB controls the genetic expression of multiple genes that participate in both inflammatory processes and immune system functions. The nuclear translocation of NF-κB occurs after oxidative stress or metabolic stimuli activate the protein, which then drives the production of pro-inflammatory cytokines, including tumor necrosis factor-α, interleukin-6, and interleukin-1β. The mitogen-activated protein kinase (MAPK) pathway functions to control the inflammatory reactions that occur because of oxidative stress. MAPK signaling activation causes inflammatory gene expression to increase while cytokines and chemokines start to be released (Figure 2). The inflammatory mediators strengthen the process of immune cell recruitment while they maintain the ongoing inflammatory state that characterizes metabolic disorders [47].

## 5. Impact of Metabolic Syndrome on Male Reproductive Function

### 5.1. Hormonal Imbalance and Hypogonadism

Men require hormonal balance because it carries vital importance for their metabolic functions and reproductive abilities. The hypothalamic–pituitary–gonadal axis functions as the primary system that controls male reproductive hormones through its complex endocrine structure that manages the release of gonadotropins and sex hormones [48]. The hypothalamus sends out gonadotropin-releasing hormone (GnRH), which causes the pituitary gland to release luteinizing hormone (LH) and follicle-stimulating hormone (FSH) into the bloodstream [49]. Elevated circulating pro-inflammatory cytokines, particularly tumor necrosis factor-α (TNF-α), interleukin-1β (IL-1β), and interleukin-6 (IL-6), have been shown to interfere with multiple levels of the reproductive axis. At the hypothalamic level, these cytokines suppress gonadotropin-releasing hormone (GnRH) secretion and alter its pulsatile release. Reduced GnRH stimulation subsequently impairs pituitary secretion of luteinizing hormone (LH) and follicle-stimulating hormone (FSH), leading to inadequate stimulation of the testes.

**Figure 3:**
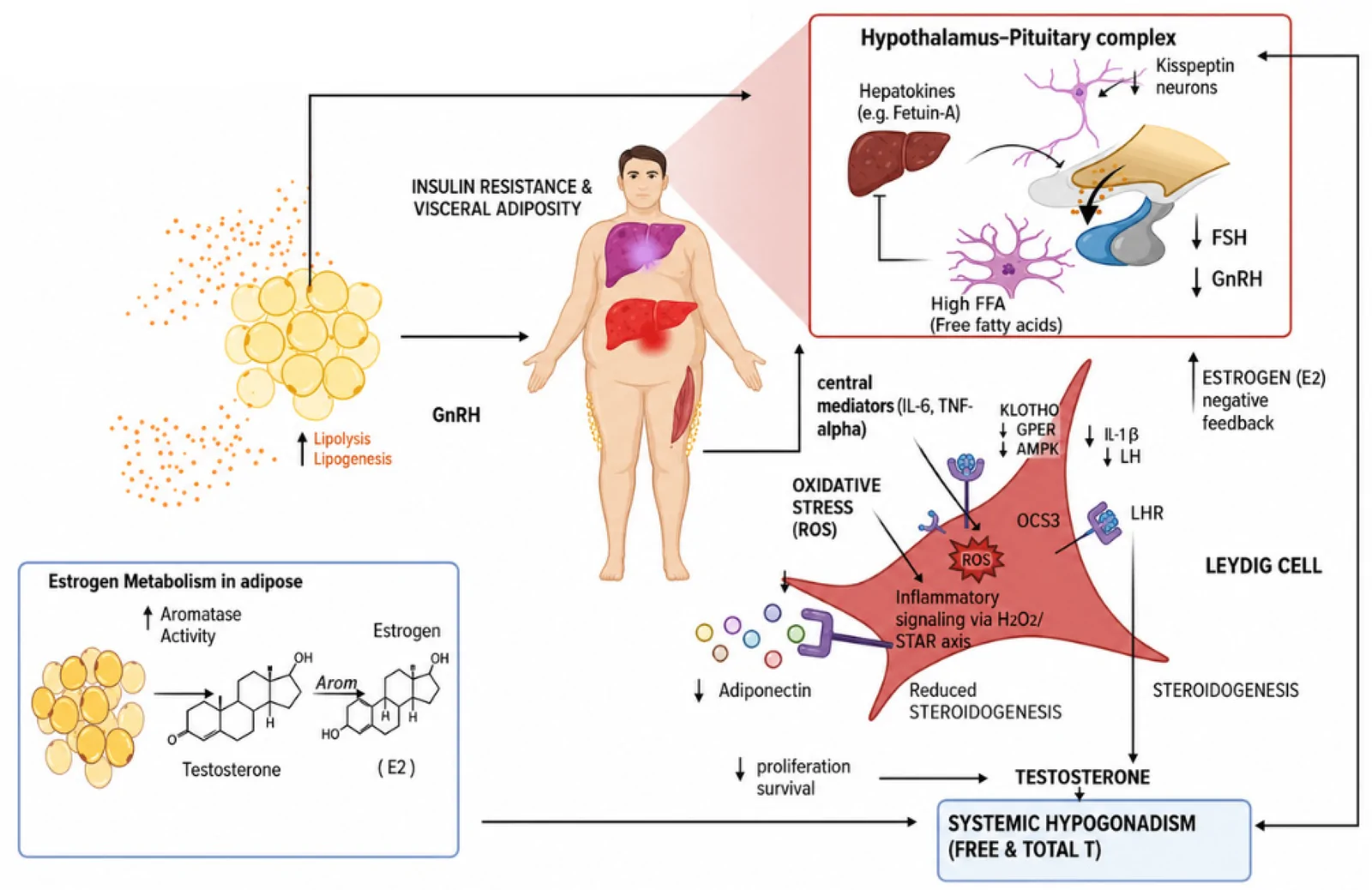
Molecular mechanism of hormonal dysregulation and hypogonadism in men with metabolic syndrome.

Chronic inflammation associated with metabolic syndrome exerts direct and indirect detrimental effects on male reproductive function through multiple cytokine-mediated mechanisms. Tumor necrosis factor-α (TNF-α) suppresses steroidogenic acute regulatory (StAR) protein expression and inhibits steroidogenic enzymes in Leydig cells, thereby reducing testosterone biosynthesis [50]. Interleukin-1β (IL-1β) suppresses hypothalamic GnRH secretion and attenuates pituitary LH release, whereas IL-6 inhibits testosterone synthesis, promotes oxidative stress, and activates the JAK/STAT3 signaling pathway within the testis [51]. Elevated cytokine concentrations also impair Sertoli cell metabolism, reduce lactate production required for germ-cell maturation, and disrupt the integrity of the blood–testis barrier by altering tight junction proteins such as occludin, claudin-11, and zonula occludens-1 [50]. Increased barrier permeability facilitates inflammatory cell infiltration into seminiferous tubules, exposing germ cells to immune-mediated injury. Furthermore, inflammatory cytokines stimulate excessive reactive oxygen species generation through activation of NADPH oxidase and mitochondrial dysfunction, resulting in lipid peroxidation, sperm DNA fragmentation, and activation of intrinsic apoptotic pathways involving Bax, cytochrome c, and caspase-3 [52]. Collectively, these cytokine-driven events impair spermatogenesis, decrease sperm concentration and motility, increase abnormal sperm morphology, and ultimately contribute to infertility in men with metabolic syndrome [53]. In obese men, excessive adipose tissue increases aromatase activity, promoting the peripheral conversion of testosterone to estradiol. Elevated estradiol exerts negative feedback on the hypothalamus and pituitary, suppressing GnRH, LH, and FSH secretion and reducing testosterone production [49]. Recent endocrine studies indicate that obesity-associated hypogonadism is characterized predominantly by reduced LH pulse amplitude, whereas alterations in GnRH pulse frequency remain less consistently demonstrated in humans because GnRH secretion cannot be measured directly in clinical settings [48]. The hormones that the testis receives from the body work together to increase Leydig cell production of testosterone while they also help the seminiferous tubules develop sperm. The hormonal balance in individuals with metabolic syndrome and obesity conditions gets disturbed because of their medical condition, which results in testosterone level reductions and testicular function loss through hypogonadism [54]. The fat tissue of obese people produces excessive aromatase enzyme activity, which serves as a primary pathway for their body hormones to become imbalanced (Figure 3). The enzyme aromatase functions to transform androgens into estrogens, with its main substrate being testosterone. The body produces more estrogen because of excess fat tissue, which leads to lower testosterone levels in the bloodstream [55]. Excess adipose tissue contributes to alterations in androgen homeostasis through multiple interconnected mechanisms. In obese men, increased aromatase activity within adipose tissue enhances the conversion of testosterone to estradiol, which may exert negative feedback on the hypothalamic–pituitary–gonadal (HPG) axis and suppress gonadotropin secretion. In addition, obesity-associated insulin resistance, chronic low-grade inflammation, elevated leptin concentrations, and reduced sex hormone-binding globulin (SHBG) levels collectively contribute to decreased circulating testosterone concentrations. Sex hormone-binding globulin (SHBG) is a glycoprotein synthesized predominantly by the liver that regulates the transport, distribution, and bioavailability of circulating sex steroids, particularly testosterone and estradiol. Approximately 40–60% of circulating testosterone is tightly bound to SHBG, whereas only the free and albumin-bound fractions are biologically active and readily available to target tissues. Consequently, SHBG plays a critical role in maintaining androgen homeostasis and interpreting serum testosterone concentrations. Men with obesity and metabolic syndrome consistently exhibit reduced SHBG concentrations, which represent one of the earliest endocrine abnormalities associated with metabolic dysfunction [52].

Hyperinsulinemia is thought to indirectly reduce hepatic sex hormone-binding globulin (SHBG) synthesis by modulating the activity of the transcription factor hepatocyte nuclear factor-4 alpha (HNF4α), resulting in decreased circulating SHBG concentrations and reduced testosterone bioavailability. This indirect mechanism contributes to the hormonal disturbances associated with obesity and metabolic syndrome. Adipokine imbalance, particularly hyperleptinemia and reduced adiponectin levels, also contributes to altered SHBG regulation [56]. As SHBG concentrations decline, total serum testosterone decreases because a smaller proportion of circulating testosterone remains protein-bound. Although free testosterone may initially remain within the normal range, progressive impairment of Leydig cell steroidogenesis ultimately results in reductions in both total and free testosterone, leading to functional hypogonadotropic hypogonadism [56].

Beyond its role as a carrier protein, SHBG has emerged as an independent biomarker of metabolic health. Low circulating SHBG concentrations are strongly associated with insulin resistance, type 2 diabetes mellitus, metabolic syndrome, cardiovascular disease, and increased systemic inflammation, independent of testosterone concentrations [57]. Clinical studies have demonstrated that reduced SHBG is associated with impaired semen quality, decreased sperm concentration and motility, and poorer reproductive outcomes in men with obesity. Therefore, simultaneous assessment of SHBG together with total and free testosterone is recommended during the endocrine evaluation of infertile men with metabolic syndrome, since measurement of total testosterone alone may underestimate clinically significant androgen deficiency [58]. Pro-inflammatory cytokines such as tumor necrosis factor-α (TNF-α) and interleukin-6 (IL-6) can impair Leydig cell steroidogenesis and disrupt HPG-axis signaling, further aggravating hypogonadism [59]. However, the relationship between adiposity and testosterone deficiency is complex and not solely attributable to increased estrogen production. The combined effects of endocrine, metabolic, and inflammatory disturbances ultimately impair spermatogenesis and contribute to reduced male fertility potential. Beyond its metabolic actions, insulin functions as an important neuroendocrine regulator of the hypothalamic–pituitary–gonadal (HPG) axis. Insulin receptors are expressed on hypothalamic neurons, including gonadotropin-releasing hormone (GnRH) neurons and kisspeptin neurons, where insulin signaling contributes to the regulation of reproductive hormone secretion. Experimental studies have demonstrated that impaired insulin signaling reduces hypothalamic GnRH release and attenuates pituitary luteinizing hormone (LH) secretion, thereby decreasing Leydig cell testosterone production [60]. Chronic insulin resistance and hyperinsulinemia further exacerbate hypothalamic inflammation and leptin resistance, disrupting normal GnRH neuronal activity and reducing LH pulse amplitude, a hallmark of obesity-associated functional hypogonadotropic hypogonadism [61]. These findings indicate that insulin resistance contributes to male hypogonadism not only through impaired testicular function but also by disrupting central neuroendocrine regulation of the HPG axis.

### 5.2. Effects on Spermatogenesis

Experimental and clinical studies provide compelling evidence that insulin resistance directly impairs spermatogenesis through endocrine and cellular mechanisms. Insulin receptors are expressed on Sertoli cells, Leydig cells, and developing germ cells, indicating that insulin signaling is essential for normal testicular function. In mouse models with selective disruption of insulin receptor signaling, impaired glucose utilization by Sertoli cells reduced lactate production, the principal energy substrate required for germ-cell development, resulting in defective spermatogenesis and decreased sperm production [62]. Clinical studies have similarly demonstrated that men with insulin resistance or type 2 diabetes exhibit significantly lower sperm concentration, progressive motility, and normal morphology than metabolically healthy controls, accompanied by increased sperm DNA fragmentation and oxidative stress [63].

Hyperinsulinemia also contributes indirectly to impaired spermatogenesis by suppressing hepatic sex hormone-binding globulin (SHBG) synthesis, reducing testosterone bioavailability, and disrupting hypothalamic–pituitary–gonadal axis function. In addition, insulin resistance promotes chronic activation of inflammatory pathways, including NF-κB and JNK, leading to increased production of TNF-α, IL-6, and reactive oxygen species within the testes [64]. These inflammatory mediators impair mitochondrial function, activate intrinsic apoptotic pathways involving Bax, cytochrome c, and caspase-3, and disrupt the blood–testis barrier, thereby compromising germ-cell survival and sperm maturation [65]. Collectively, these experimental and clinical observations demonstrate that insulin resistance contributes to male infertility through both direct metabolic impairment of testicular cells and indirect endocrine and inflammatory mechanisms.

Clinical studies provide complementary evidence that obesity-associated insulin resistance is associated with impaired reproductive outcomes. Men with metabolic syndrome consistently exhibit lower sperm concentration, progressive motility, and normal morphology, together with increased sperm DNA fragmentation and reduced fertilization potential compared with metabolically healthy men [66]. Collectively, these experimental and clinical findings indicate that insulin resistance impairs spermatogenesis through direct metabolic effects on Sertoli cells, disruption of the blood–testis barrier, oxidative stress, and inflammatory injury, thereby contributing significantly to male infertility.

### 5.3. Alterations in Semen Parameters

A growing body of clinical evidence demonstrates that obesity and metabolic syndrome are associated with significant deterioration of semen quality. One of the first systematic reviews by Santi D et al. reported that metabolic syndrome and obesity are strongly associated with impaired male reproductive function, including reduced testosterone levels, poor semen quality, and increased risk of infertility [67].

Clinical investigations have similarly shown that men with metabolic syndrome exhibit significantly lower sperm concentration, progressive motility, total motile sperm count, and normal sperm morphology compared with metabolically healthy controls. In a multicenter observational study, Semen parameters serve as essential diagnostic tools to assess male reproductive health and fertility capability. The parameters consist of seven components: sperm concentration, motility, morphology, semen volume, vitality, and sperm DNA integrity [68]. The metabolic disorders of obesity and metabolic syndrome lead to multiple pathways that cause metabolic dysfunction, oxidative stress, chronic inflammation, and hormonal imbalance to produce harmful effects on semen quality [69]. The changes that occur in these processes lead to harmful effects that restrict sperm development and maturation and operational capacity, which heighten the probability of male infertility [70]. The testicular cells lose their capability to function properly because of insulin resistance combined with metabolic disorders, which lead to decreased sperm production. Metabolic disorders affect sperm motility, which describes how well sperm can reach the oocyte through movement [71]. The research demonstrates that both obesity and metabolic syndrome result in reduced progressive sperm motility. Sperm cells experience energy production problems because of increasing oxidative stress and mitochondrial dysfunction, which serve as crucial energy for their movement. The reduced ability of sperm to move results in their decreased capacity to reach and fertilize the egg [72].

### 5.4. Testicular Microenvironment and Cellular Damage

The testicular microenvironment maintains normal spermatogenesis and male reproductive function through its essential functions. The testicular microenvironment suffers disruption from metabolic disorders, including obesity and metabolic syndrome, which result in cellular damage and reproductive function impairment [73]. The blood–testis barrier (BTB) protects the testicular microenvironment, which suffers from metabolic disorders. The BTB exists to protect developing germ cells through its tight junctions, which Sertoli cells use to create a barrier against dangerous substances and immune system attacks. The barrier gets weakened by chronic inflammation and oxidative stress, which permits harmful substances and immune cells to invade the seminiferous tubules. Immune system disruption leads to germ cell damage, which further prevents spermatogenesis [74]. In clinical practice, many other sources of oxidative stress and inflammation have been noted—lifestyle (tobacco and alcohol use) and other clinical conditions related to male infertility, e.g., varicocele, leukocytospermia, Sexually Transmitted Infections (STIs), HIV, and bacterial prostatitis.

Metabolic syndrome impacts testicular blood circulation and nutrient delivery to the testis. The metabolic disorders that cause vascular dysfunction and endothelial damage lead to decreased blood circulation toward the testis, which prevents proper oxygen and essential nutrients from reaching germ cell development [75]. Testicular function depends on microcirculation, which provides the metabolic support needed for normal testicular function. Obesity raises scrotal temperature, which creates adverse effects on the testicular microenvironment. Excess scrotal adipose tissue causes local temperature increases, which disrupt the temperature-sensitive spermatogenesis process. Germ cells experience apoptosis while sperm production decreases from even slight scrotal temperature increases [76]. Recent advances in proteomics have provided novel insights into the molecular mechanisms underlying oxidative stress-mediated male infertility. Proteomic analyses of seminal plasma and spermatozoa have identified alterations in proteins involved in energy metabolism, antioxidant defense, apoptosis, and sperm function. These approaches have been particularly valuable in understanding idiopathic male infertility and may facilitate the discovery of novel biomarkers and personalized therapeutic strategies for men with reproductive dysfunction.

## 6. Clinical Evidence Linking Metabolic Syndrome with Male Infertility

Clinical trials and cohort studies provide essential evidence that shows how metabolic disorders affect male reproductive health. The research demonstrates how obesity, insulin resistance, and metabolic syndrome conditions impact semen quality, hormonal balance, and fertility outcomes. The study designs enable researchers to examine both relationships between variables and the effects of medical and lifestyle changes on reproductive health [77]. Cohort studies track a selected group of people throughout an extended period to study how particular health risks, including obesity and metabolic disorders, affect their health outcomes. Cohort studies assess male fertility through testing reproductive characteristics of men who possess various metabolic conditions [78]. Cohort studies investigate how type 2 diabetes affects male reproductive health. Research shows that diabetic men display hormonal disturbances with decreased testosterone levels and increased estrogen levels, which disrupt their ability to produce sperm [79]. The reproductive system suffers from long-term metabolic disorders because they create oxidative stress together with inflammation, which results in harm to both sperm cells and testicular tissue.

Clinical trials deliver stronger evidence than observational cohort studies because they assess specific interventions that aim to enhance metabolic health and reproductive outcomes. The research studies evaluate how weight-loss programs, dietary changes, exercise, and drug treatments impact semen quality and hormone levels [80]. Clinical trials provide important evidence regarding the safety, efficacy, optimal dosing, and long-term outcomes of therapeutic interventions for obesity-associated metabolic syndrome and male infertility. They are essential for validating promising preclinical findings, assessing treatment effectiveness in diverse patient populations, and facilitating the translation of research into evidence-based clinical practice. Researchers investigate antioxidant supplements as a potential treatment to lower oxidative stress levels in men who suffer from metabolic disorders through clinical trial research. Researchers evaluated the effects of vitamin C and E supplements together with coenzyme Q10 and zinc and selenium on sperm quality and DNA damage reduction [81]. These antioxidants are believed to counteract excessive reactive oxygen species generation, reduce lipid peroxidation, and protect sperm DNA from oxidative damage. Clinical investigations have reported improvements in selected semen parameters, such as sperm concentration, motility, morphology, and DNA integrity, following antioxidant therapy. Coenzyme Q10 has been associated with enhanced mitochondrial function and sperm motility, whereas zinc and selenium contribute to spermatogenesis and antioxidant enzyme activity. Vitamins C and E act synergistically to neutralize free radicals and preserve membrane stability. Omega-3 fatty acids have also been shown to improve sperm membrane fluidity and overall sperm function. However, the available evidence remains heterogeneous, and several systematic reviews have suggested that the beneficial effects of antioxidant supplementation are modest and inconsistent across studies. Therefore, although antioxidant therapy may provide supportive benefits in selected patients with oxidative stress-related infertility, further large-scale randomized controlled trials are required to establish optimal treatment regimens and confirm their long-term effectiveness (Table 1).

Interventional studies have demonstrated that lifestyle modifications, weight reduction, dietary interventions, physical activity, and pharmacological therapies can improve metabolic health and partially restore reproductive function in men with obesity and metabolic syndrome. These findings highlight the importance of early intervention and multidisciplinary management to improve hormonal balance, semen quality, and fertility outcomes [82]. This variability likely reflects differences in patient characteristics, severity of metabolic dysfunction, duration of intervention, and the multifactorial nature of male infertility. Since reproductive outcomes are influenced by numerous confounding factors, including age, genetic predisposition, comorbidities, environmental exposures, and lifestyle habits, further large-scale, long-term, and well-controlled clinical studies are required to establish definitive causal relationships and optimize therapeutic strategies [83].

**Table 1:** Observational studies evaluating the association between metabolic syndrome and male reproductive function.

Study	Study Design	Population	Main Findings
Salvio et al., 2022 [84]	Cross-sectional cohort	1337 infertile men	Lower testosterone, SHBG, inhibin B; increased hypogonadism prevalence
Serbouti et al., 2026 [85]	Cross-sectional study	8395 reproductive-age men	Reduced sperm morphology and progressive motility
Belladelli et al., 2022 [86]	Case–control pilot	54 men with and without MetS	Lower sperm concentration, motility, vitality; increased DNA fragmentation
Le et al., 2021 [87]	Prospective cohort	744 infertile men with prediabetes	Lower testosterone and SHBG; higher sperm DNA fragmentation
Avcı et al., 2025 [88]	Cross-sectional cohort	726 infertile men	Insulin resistance associated with poorer semen and hormonal parameters
Darand et al., 2023 [89]	Cross-sectional study	534 men from infertile couples	Metabolic markers correlated with semen abnormalities
Dena et al., 2025 [90]	Observational study	518 infertile men	Lower serum testosterone in MetS group
Foster-Schubert et al., 2012 [91]	Cross-sectional study	218 infertile men	Increased BMI associated with abnormal sperm morphology
Ryan et al., 2017 [92]	Prospective cohort	32 diabetic men	Reduced sperm motility and mitochondrial dysfunction

The available epidemiological evidence consistently demonstrates that the adverse effects of obesity and metabolic syndrome on male fertility are clinically meaningful rather than merely statistically significant. Population-based cohort studies indicate that increasing body mass index is associated with a progressive decline in reproductive hormone concentrations and semen quality, while the coexistence of multiple metabolic abnormalities further amplifies infertility risk [67]. Meta-analyses have shown that metabolic syndrome is associated with significant reductions in sperm concentration, total sperm count, progressive motility, and normal morphology, accompanied by increased oxidative stress and sperm DNA fragmentation. Moreover, obese men are considerably more likely to develop functional hypogonadism characterized by low circulating testosterone concentrations and impaired hypothalamic–pituitary–gonadal axis activity. Collectively, these findings support the concept that obesity and metabolic syndrome should be regarded as independent and modifiable risk factors for male infertility, emphasizing the importance of early metabolic screening and lifestyle intervention in men presenting with reproductive dysfunction [72].

Several review articles have synthesized findings from observational and clinical studies, consistently concluding that metabolic syndrome adversely affects male reproductive hormones, semen quality, and fertility outcomes [70].

Clinical evidence further supports the strong association between metabolic dysfunction and male hypogonadism in a large cross-sectional study of men with type 2 diabetes. The study also demonstrated significant associations between obesity, insulin resistance, poor glycemic control, and reduced testosterone concentrations, suggesting that metabolic dysfunction contributes to suppression of the hypothalamic–pituitary–gonadal axis rather than primary testicular failure. These findings support the concept that hypogonadotropic hypogonadism is a common endocrine complication of metabolic syndrome and type 2 diabetes and may contribute to impaired fertility and reproductive dysfunction [74].

## 7. Preventive and Therapeutic Strategies

### 7.1. Lifestyle Modifications and Weight Management

The combination of dietary changes with increased physical activity and weight loss and new behavioral patterns leads to improvements in metabolic health and reproductive success. The most successful method for weight loss requires people to consume a diet that contains controlled calories from diverse food sources [93]. Research demonstrates that when overweight or obese men experience moderate weight loss, they will show increases in testosterone levels, and their sperm concentration will rise while their sperm motility will improve [94]. The dietary quality of a person directly impacts their metabolic outcomes and their ability to reproduce. People who consume diets that contain high amounts of fruits, vegetables, whole grains, and lean proteins, along with healthy fats, will receive vital nutrients that provide antioxidants that help their cells operate, and their sperm stay safe from oxidative harm [95]. The combination of vitamin C, vitamin E, zinc, selenium, and omega-3 fatty acids brings about better protection against oxidative damage, which results in improvement in sperm functionality. The combination of high processed foods intake together with saturated fat consumption and refined sugar consumption leads to greater oxidative stress and inflammation, which results in worse semen quality [96]. People need to make lifestyle changes that will treat their behavioral risk factors because these factors will increase their chances of experiencing metabolic dysfunction and infertility problems. Studies show that smoking, heavy alcohol use, and chronic stress lead to lower semen quality and hormonal changes [97]. Smoking brings toxic substances into the body, which causes oxidative stress and DNA damage to sperm cells. Weight loss has been consistently associated with increased total and free testosterone concentrations, higher sex hormone-binding globulin (SHBG) levels, and partial restoration of hypothalamic–pituitary–gonadal axis function. Practicing yoga, meditation, and aerobic exercise lower the oxidative stress, increase pelvic blood flow, and alleviate psychological stress, which all contribute to better sperm quality, DNA integrity, and fertility in metabolic syndrome–associated infertility.

### 7.2. Nutritional Interventions and Antioxidant Therapy

The combination of nutritional interventions with antioxidant therapy results in enhanced metabolic health and improved male reproductive capability for people who suffer from multiple medical conditions including obesity, insulin resistance, and metabolic syndrome [98]. Whole food diets that contain fruits and vegetables, whole grains and legumes, lean proteins and healthy fats provide essential vitamins and minerals together with dietary compounds that enhance both metabolic function and reproductive performance. The foods contain natural antioxidants that protect the body by neutralizing reactive oxygen species (ROS) that cause sperm membrane, protein, and DNA damage [99]. The diet delivers multiple antioxidants together with anti-inflammatory substances and beneficial unsaturated fats that include omega-3 fatty acids. Studies indicate that following such dietary patterns leads to better semen parameters, which include higher sperm concentration and improved motility together with better metabolic indicators [100]. Antioxidant therapy serves as a standard treatment that doctors use to help men with infertility conditions or metabolic disorders who need to decrease their oxidative stress levels.

### 7.3. Pharmacological Approaches

Pharmacological approaches are established treatments following lifestyle and nutritional methods when they fail to resolve metabolic disorders that affect male reproductive health. The drugs activate the hypothalamic–pituitary–gonadal system to induce natural testosterone production. The treatments restore hormonal balance, which leads to improved spermatogenesis and better semen quality [101]. Aromatase inhibitors have been investigated as a potential therapeutic option for selected men with obesity-associated functional hypogonadism because they reduce the peripheral conversion of testosterone to estradiol, thereby increasing circulating testosterone concentrations and, in some cases, improving gonadotropin secretion [102]. However, their routine use remains controversial [103]. Estrogens play indispensable physiological roles in men, including the maintenance of bone mineral density, regulation of hepatic lipid metabolism, modulation of adipose tissue distribution, cardiovascular function, and preservation of libido and sexual health [104]. Consequently, excessive suppression of estrogen synthesis may adversely affect skeletal health and other metabolic processes. Current evidence therefore suggests that aromatase inhibitors should not be considered routine therapy for obesity-associated hypogonadism but may be appropriate only in carefully selected patients after comprehensive endocrine evaluation [105]. Long-term randomized clinical trials are still needed to establish their efficacy and safety in men with metabolic syndrome and infertility [106].

### 7.4. Hormonal Therapy and Metabolic Control

The two main medical approaches for treating male reproductive dysfunction that result from metabolic disorders, which include obesity, insulin resistance, and metabolic syndrome, require both hormonal therapy and metabolic control. The conditions result in hormonal disturbances through which testosterone and estrogen levels become unbalanced, and this condition hinders the process of sperm production and decreases the quality of semen [107]. Endocrine disorders need correction through hormonal therapies, which will produce better metabolic control to restore reproductive capabilities. High estrogen levels are associated with reduced spermatogenesis due to suppression of the hypothalamic–pituitary–gonadal axis and impaired testicular function [108]. Several therapeutic agents exert their effects by antagonizing estrogen receptors or suppressing estrogen biosynthesis, thereby re-establishing the androgen–estrogen balance. Hormonal therapy is often integrated with metabolic control strategies, since metabolic dysfunction can directly influence endocrine regulation [109]. Improving insulin sensitivity and reducing adiposity can help normalize hormonal signaling within the HPG axis. The process of effective metabolic control requires people to make dietary changes, engage in regular physical activity, manage their weight, and receive medical treatment for conditions like diabetes and dyslipidemia [110]. The body can achieve better metabolic control, which leads to decreased inflammation and oxidative stress that damage testicular function and hinder sperm development. The body may achieve improved glucose control together with better lipid balance, which enhances blood circulation and nutrient delivery to the testis, creating a healthier testicular microenvironment [111]. Men with clinically significant hypogonadism who do not want to have children can use testosterone replacement therapy as a treatment option. The treatment of testosterone replacement therapy allows patients to experience relief from three different symptoms, which include fatigue, reduced libido, and decreased muscle mass [112]. GLP-1 receptor agonists, which have been shown to be useful in weight loss and metabolic disorders, could also be useful to male fertility as they have been found to preserve sperm motility, and viability, but larger clinical trials are required [113,114].

### 7.5. Assisted Reproductive Technologies 

Assisted Reproductive Technologies (ART) function as essential treatment options for people who experience infertility due to metabolic syndrome when their metabolic and hormonal problems prevent them from conceiving naturally [115]. ART consists of medical procedures that help both fertilization and embryo development to occur [116]. The procedures include intrauterine insemination (IUI), in vitro fertilization (IVF), and intracytoplasmic sperm injection (ICSI). The selection of a method relies on three factors, which include the degree of male infertility and the results of semen analysis and the reproductive capabilities of both partners. Intracytoplasmic sperm injection (ICSI) represents one of the most frequently applied methods to treat male infertility [117]. The procedure involves direct sperm injection into the cytoplasm of an oocyte, which allows the process to bypass numerous natural fertilization barriers. Clinical studies suggest that men with obesity and metabolic syndrome may experience significant improvements in reproductive endocrine function following interventions that improve metabolic health, particularly lifestyle modification, weight reduction, and optimization of insulin sensitivity [118,119]. Several intervention studies have also reported improvements in sperm concentration, progressive motility, and overall semen quality after sustained weight reduction, although the magnitude of improvement varies among individuals and depends on the severity and duration of metabolic dysfunction [120].

## 8. Future Research Directions

### 8.1. Emerging Biomarkers for Early Detection

Early detection of reproductive dysfunction, which occurs together with metabolic disorders, serves as the primary method for stopping all future fertility issues while also providing necessary medical treatments at the correct time. Recent research efforts aim to discover new biomarkers that can reveal initial metabolic and reproductive health changes before both semen parameters and hormonal balance show major deviations from normal levels [121]. The group of biomarkers includes molecular features together with biochemical, genetic, and inflammatory markers that show changes that occur in human metabolic systems and oxidative stress levels and testicular activity. The group of emerging biomarkers includes inflammatory markers that show a connection with metabolic syndrome [122]. Individuals with obesity and insulin resistance present two main symptoms that define their condition as chronic low-grade inflammation. The metabolic dysfunction experienced by people results in elevated levels of CRP, TNF-α, and interleukin-6 (IL-6) [123]. The testicular microenvironment experiences damage from early inflammatory changes, which become evident through rising marker levels that pose a threat to Sertoli cell activity and spermatogenesis [124].

Researchers have found that oxidative stress biomarkers make up a second research area that shows potential for future exploration. The process of oxidative stress damage leads to two consequences that affect sperm performance and result in DNA damage. Scientists now use three biomarkers, including malondialdehyde (MDA), reactive oxygen species (ROS), and total antioxidant capacity (TAC), to study oxidative balance through the analysis of seminal plasma or blood samples [125]. Men with metabolic disorders show early reproductive problems when they exhibit high oxidative stress levels together with low antioxidant protection. The evaluation of hormonal biomarkers provides critical information that assists in the identification of initial symptoms [126]. The measurement of testosterone levels, together with luteinizing hormone (LH), follicle-stimulating hormone (FSH), sex hormone-binding globulin (SHBG), and estradiol levels, enables researchers to assess disruptions across the hypothalamic–pituitary–gonadal system. The tiniest hormonal fluctuations reveal to doctors the initial signs that point to metabolic-induced hypogonadism and spermatogenesis problems [127].

The research findings from the latest studies demonstrate that adipokines, which act as hormones from adipose tissue, serve as essential metabolic controllers that link to inflammatory processes. The molecules, which include leptin and adiponectin, act as vital components for controlling metabolic processes and inflammatory responses [128]. The hormonal signaling system that controls reproductive functions needs to maintain normal adipokine levels because abnormal levels of these adipokines disrupt this system. The research shows that obesity results in raised leptin levels, which negatively affect testosterone production and sperm quality [129]. Although leptin normally functions as a regulator of appetite and energy homeostasis, chronic hyperleptinemia in obese individuals may exert detrimental effects on male reproductive function. Excess leptin can disrupt hypothalamic–pituitary–testicular axis signaling by impairing gonadotropin-releasing hormone (GnRH) secretion and altering luteinizing hormone (LH) production, ultimately reducing Leydig cell stimulation and testosterone synthesis.

Elevated leptin levels are also associated with increased oxidative stress, chronic low-grade inflammation, and impaired Sertoli cell function, thereby creating an unfavorable environment for spermatogenesis. Experimental and clinical studies have demonstrated associations between hyperleptinemia and reduced sperm concentration, motility, viability, and increased sperm DNA fragmentation. The advancements in molecular biology research have enabled scientists to identify genetic and epigenetic biomarkers that affect male fertility. The combination of changes in DNA methylation patterns, microRNA (miRNA) expression, and sperm chromatin structure enables scientists to detect metabolic stress affecting reproductive cells at an early stage [130]. MicroRNAs function as essential components for gene regulation, which controls spermatogenesis and inflammation, and metabolic processes, making them effective diagnostic tools. The new research field investigates the use of metabolomic biomarkers, which study the metabolic byproducts found in biological fluids like blood and seminal plasma [131]. The analysis of metabolomic data enables scientists to detect reproductive health problems that stem from altered energy metabolism, lipid metabolism, and oxidative pathway changes. The researchers are studying proteomic biomarkers to find specific proteins that play a role in sperm development and testicular function. The proteomic study of seminal plasma shows how protein alterations affect sperm movement and development and immune defense mechanisms, which creates new possibilities for discovering metabolic syndrome-related fertility problems in men [132]. The clinical application of these biomarkers will enhance the early detection and treatment of male infertility cases that stem from metabolic disorders. Clinicians can use early detection of metabolic and reproductive disorders to start appropriate treatments, which include lifestyle changes and nutritional programs and medical solutions before serious reproductive harm develops [133].

### 8.2. Molecular Targets for Therapeutic Intervention

The metabolic disorder known as metabolic syndrome includes insulin resistance, which prevents cells from processing energy inside various body parts, including the testes. Leydig cells, Sertoli cells, and developing germ cells rely on adequate energy supply and insulin-mediated metabolic pathways for normal steroidogenesis and spermatogenesis. Disruption of these pathways results in impaired glucose utilization, mitochondrial dysfunction, and increased oxidative stress within the testicular microenvironment. Furthermore, insulin resistance is accompanied by chronic low-grade inflammation and elevated circulating cytokines, which can interfere with testicular function and hormonal regulation. The pathway contains insulin receptors and insulin receptor substrates (IRS) and the PI3K–Akt signaling pathway, which control the processes of glucose uptake and cellular growth and survival. Scientists need to develop new treatment methods that will enhance insulin sensitivity together with insulin signaling because these methods will help testicular cells restore their normal metabolic functions and support the development of sperm [134]. The second key objective targets AMP-activated protein kinase (AMPK), which functions as a cellular energy sensor that controls metabolic processes. The energy equilibrium maintenance system of AMPK operates through its three functions, which include boosting glucose uptake, promoting fatty acid oxidation, and increasing mitochondrial activity [135]. The activation of AMPK leads to better metabolic function and decreased inflammation. The study focuses on two molecular targets, including inflammatory signaling pathways. The reproductive system is disrupted by chronic low-grade inflammation from obesity and metabolic syndrome, which damages testicular tissue [136]. NF-κB, together with various cytokine signaling pathways, operates as the fundamental mechanism that regulates inflammatory processes. The testicular microenvironment receives protection from therapeutic agents that block NF-κB activation and reduce inflammatory cytokine production, thus safeguarding testicular cells from damage [137].

### 8.3. Role of Personalized Medicine in Male Infertility

Personalized medicine enables better diagnostic methods and treatment plans and reproductive results for male infertility cases that involve metabolic disorders like obesity, insulin resistance, and metabolic syndrome by using personalized treatment approaches that study each patient’s distinct biological characteristics [138]. The fundamental element of personalized medicine involves conducting genetic and genomic assessments of patients. Scientists have developed molecular biology techniques that enable them to discover genetic variations that impact reproductive function, hormone regulation, and metabolic activities [139].

Metabolic profiling shows multiple metabolic pathways through its assessment of insulin levels, lipid profiles, inflammatory markers, and hormone levels. The assessment of these metabolic markers enables clinicians to determine which metabolic disorders are responsible for male reproductive health issues [140]. The patient’s metabolic profile determines which dietary interventions and exercise programs, and pharmacological therapies and hormonal treatments will be included in their personal treatment plan. Biomarker-based diagnosis also plays a significant role in personalized medicine [141]. The new biomarkers, which include oxidative stress indicators, inflammatory cytokines and adipokines, and sperm DNA fragmentation markers, enable researchers to study the complete reproductive system performance. The use of these biomarkers enables doctors to identify initial signs of reproductive problems, which help them choose treatments that solve known biological issues [142]. The research field of pharmacogenomics studies the way an individual’s genetic makeup determines their medication response. Genetic variations that affect drug metabolism and receptor sensitivity make some men respond differently to hormonal therapies, insulin-sensitizing drugs, and antioxidant treatments. The development of personalized pharmacological strategies enables treatment solutions that enhance effectiveness while decreasing potential adverse effects.

## 9. Conclusions

Accumulating evidence from epidemiological studies, clinical investigations, and experimental models demonstrates that obesity and metabolic syndrome adversely affect male reproductive health through multiple interconnected mechanisms. Clinical studies consistently report reductions in serum testosterone concentrations, impaired hypothalamic–pituitary–gonadal axis function, deterioration of semen parameters, and increased sperm DNA fragmentation in men with metabolic syndrome. Experimental investigations further demonstrate that insulin resistance, chronic inflammation, oxidative stress, mitochondrial dysfunction, and impairment of Sertoli and Leydig cells collectively disrupt spermatogenesis and compromise male fertility. Importantly, intervention studies indicate that lifestyle modification, weight reduction, and optimization of metabolic health can partially restore reproductive hormone profiles and improve semen quality, highlighting the potential reversibility of obesity-associated reproductive dysfunction. Nevertheless, additional well-designed prospective clinical trials are needed to clarify the long-term effects of metabolic interventions on fertility outcomes and to establish evidence-based therapeutic strategies for men with obesity and metabolic syndrome.

## Data Availability

No new data were created or analyzed in this study. Data sharing is not applicable to this article.
